# White Light-Emitting Devices Based on Inorganic Perovskite and Organic Materials

**DOI:** 10.3390/molecules24040800

**Published:** 2019-02-22

**Authors:** Shuming Chen, Chen Chen, Cong Bao, Muhammad Mujahid, Ye Li, Ping Chen, Yu Duan

**Affiliations:** 1 College of Science, Changchun University of Science and Technology, Changchun 130012, China; shuming828@163.com; 2 State Key Laboratory on Integrated Optoelectronics, College of Electronic Science and Engineering, Jilin University, Changchun 130012, China; cchen18@mails.jlu.edu.cn (C.C.); baocong16@mails.jlu.edu.cn (C.B.); mujahidiqbal133@gmail.com (M.M.); pingchen@jlu.edu.cn (P.C.)

**Keywords:** inorganic perovskite, polymer, white PeLED

## Abstract

Perovskite-based materials have attracted considerable attention in photoelectric devices. In this paper, we report the one-step fabrication of spin-coated CsPbBr_2.5_I_0.5_ perovskite films doped with PAN (polyacrylonitrile) polymer. A red perovskite LED (PeLED) composite film was fabricated which featured a maximum luminance value of 657 cd/m^2^ at 8 V. We fabricated white PeLEDs by combining hole transporting layer material emission, perovskite–polymer composite material PAN:CsPbBr_2.5_I_0.5_, and pure inorganic perovskite CsPbBr_3_ as a luminescent layer. The maximum luminance of the device was 360 cd/m^2^ at 7 V, and the color coordinate was (0.31, 0.36). We obtained an ideal white light-emitting device that paves the way for further development of white PeLEDs.

## 1. Introduction

Electroluminescent devices have been the focus of many studies in the fields of physics and chemistry. In comparison to traditional inorganic semiconductors and organic small molecules [1,2,3], materials based on ABX_3_ perovskites have attracted the attention of many researchers due to their excellent photoelectric properties. State-of-the-art perovskite solar cells already feature an efficiency of 22.1%, which is comparable to photovoltaic cells made from traditional inorganic semiconductor materials [4,5,6]. Perovskite-based materials are also used as effective low-threshold gain media in optical pump lasers [7,8]. Moreover, perovskite light-emitting devices also present other advantages such as high photoluminescence quantum yield (PLQY), easy adjustment of color luminescence, and high color purity [9,10,11,12]. The excellent electronic and optical properties of these materials are ideal for high-performance optoelectronic devices and light-emitting diodes (LEDs) for displays and illumination. White light plays an important role in the fields of illumination and display, as the main light source of solid-state devices and backlights in liquid-crystal displays [13]. Therefore, research into white light-emitting perovskite devices is of great significance. Since the first room-temperature electroluminescent device manufactured in 2014, great progress has been made in the application of perovskite materials as light-emitting devices, especially for green light-emitting devices based on lead perovskite bromide (e.g., MAPbBr_3_ and CsPbBr_3_). After a relatively short development period, the luminance and external quantum efficiency (EQE) of green perovskite LEDs reached 10,000 cd/m^2^ and 10%, respectively [14]. The Snaith group first reported green and infrared perovskite light-emitting diodes (PeLEDs) with tunable band gaps via changes in the chemical composition. These PeLEDs achieved luminescence of 364 cd/m^2^ and radiation of 6.8 Wsr^−1^m^−2^ [15].

Organic–inorganic hybrid perovskite materials based on methylamine (CH_3_NH_3_) are very sensitive to moisture and heat, leading to poor stability of PeLEDs [16,17]. An effective strategy for improved heat and humidity stability of perovskite solar cells involves replacing the MA cations in the perovskite with formazan (FA) or Cs [18,19,20]. Therefore, partial or complete cation substitution can produce more stable PeLEDs. Although FA cations are more stable than MA cations, the former is still susceptible to humidity [21]. Therefore, the most stable PeLED can be obtained by substituting inorganic Cs cations for unstable organic cations [22].

In order to achieve high-efficiency PeLEDs based on perovskite thin films, dense and uniform grain and reducing defects are prerequisites for high-quality perovskite thin films, which is beneficial to enhance radiation recombination and inhibition of nonradiative recombination [23,24]. Evidently, the morphology of the perovskite layer is very important to the performance of PeLEDs. A successful protocol to achieve this involves mixing perovskite precursors with polymeric materials, such as polyethylene oxide (PEO) [25] and polyacrylonitrile (PAN) [26].

In this work, we doped polymer material PAN into inorganic perovskite CsPbBr_2.5_I_0.5_ precursor solution. High coverage and average PAN:CsPbBr_2.5_I_0.5_ mixed film was obtained by the one-step spin-coating method, and we fabricated a red perovskite device with the mixed film as the luminescent layer, and the maximum luminescence of the device was 657 cd/m^2^ at 8V. At the same time, we fabricated a multi-luminescent layer structure white PeLED by combining organic small molecule material NPB, perovskite–polymer composite material PAN:CsPbBr_2.5_I_0.5_, and pure inorganic perovskite CsPbBr_3_ as a luminescent layer. The maximum luminescence of the device was 360 cd/m^2^ at 7 V, the current efficiency was 0.2 cd/A, and the color coordinate of the PeLED was (0.31, 0.36). Although the brightness and efficiency were lower, the exciton distribution of the white PeLED under the multi-luminescent layer structure was consistent with ideal white emission.

## 2. Results and Discussion

### 2.1. Device Structure

**Figure 1:**
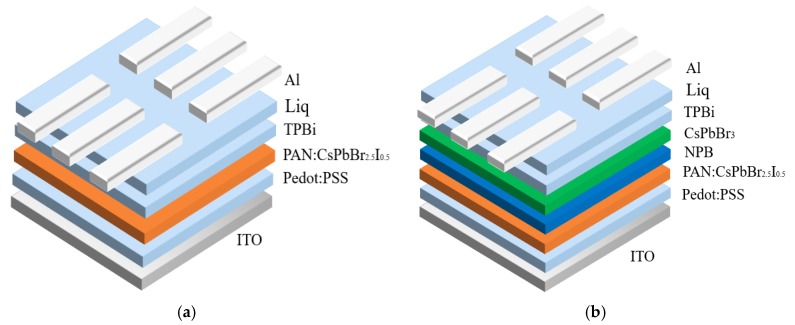

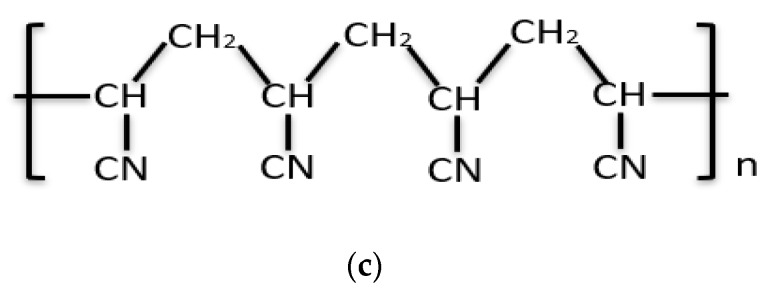
(**a**) The device structure diagram of a red perovskite light-emitting diode (PeLED); (**b**) the device structure diagram of a white PeLED; (**c**) the chemical structure of polyacrylonitrile (PAN).

Figure 1a shows a scheme of the red PeLED devices which used ITO as the anode, PEDOT: PSS as the hole transport layer (HTL), PAN:CsPbBr_2.5_I_0.5_ as the red luminescent layer, TPBi as the electron transport layer (ETL), Liq as the electron injection layer (EIL), and Al as the cathode. Figure 1b shows the structure of the white PeLED device which employed PAN:CsPbBr_2.5_I_0.5_, NPB, and CsPbBr_3_ as the red, blue, and green light-emitting layers respectively. The hole transport layer, electron transport layer, and electron injection layer of the white PeLED were the same as those in the red PeLED. Figure 1c shows the chemical structure of PAN.

### 2.2. Characterization of Perovskite Films

**Figure 2:**
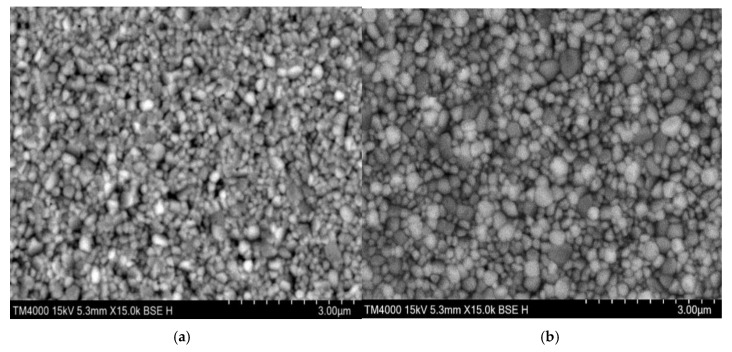
(**a**) SEM image of CsPbBr_2.5_I_0.5_ film without PAN; (**b**) SEM image of CsPbBr_2.5_I_0.5_ film with PAN.

**Figure 3:**
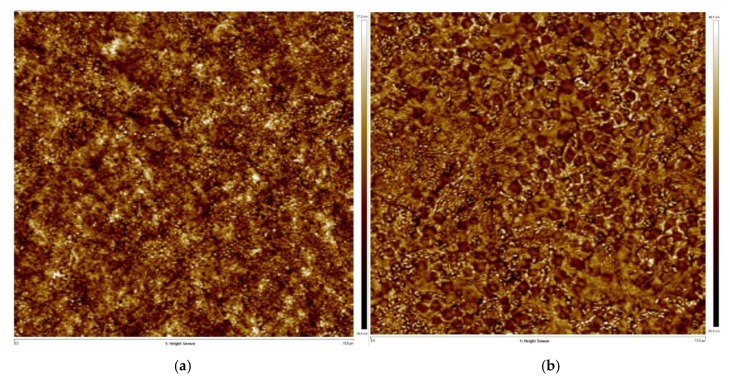
(**a**) Atomic force microscope (AFM) image of CsPbBr_2.5_I_0.5_ film without PAN; (**b**) AFM image of CsPbBr_2.5_I_0.5_ film with PAN.

The effect of PAN on the surface morphology of the light-emitting layer was investigated using scanning electron microscopy (SEM). Figure 2 shows a surface image of a perovskite film with and without PAN. The bare perovskite film featured numerous pinholes and poor coverage, which causes unnecessary nonradiative recombination [27]. The PAN:CsPbBr_2.5_I_0.5_ mixed film exhibited a continuous crystal form, a drastic reduction in the number of pinholes, and almost no voids. The coverage and uniformity were remarkably improved. Introduction of PAN caused the concomitant increase of the perovskite crystals size. Consistent with the SEM observations, atomic force microscope (AFM) images of the bare perovskite film exhibited a roughness of 6 nm (Figure 3). This high surface roughness results in an inferior interface with the NPB layer, which severely limits the performance of the PeLEDs [28]. The film coated with PAN was characterized by a roughness of 4.7 nm, which represents a considerable improvement in terms of the film surface smoothness. These results suggest that the introduction of the PAN plays an essential role in controlling the morphology of the perovskite, which means that the mixed film can be used to fabricate more efficient light-emitting devices. We used the stylus profiler to measure the thickness of CsPbBr_2.5_I_0.5_ and PAN:CsPbBr_2.5_I_0.5_ films at 2000 rpm/s and 4000 rpm/s respectively. The thickness at 2000 rpm/s was 40 nm and 45 nm, and the thickness at 4000 rpm/s was 17 nm and 20 nm. The film thickness of the mixed solution containing the polymer is slightly higher than that of the pure perovskite solution at the same rotation speed because the viscosity of the solution is increased by the introduction of the polymer. It can be seen that the film thickness is mainly determined by the spin coating speed, and the effect of the PAN addition on the film thickness is slight.

**Figure 4:**
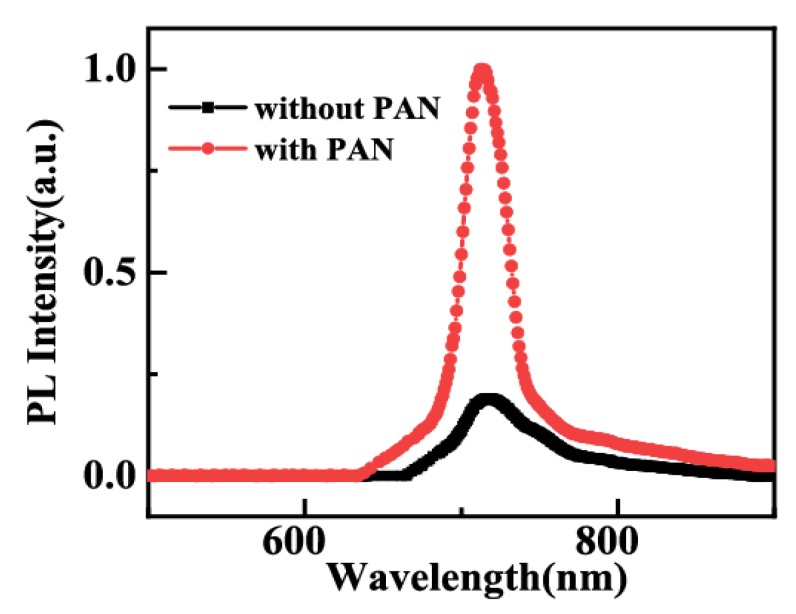
Photoluminescence spectra of the CsPbBr_2.5_I_0.5_ film.

Figure 4 shows the photoluminescence (PL) spectra used to evaluate the optical properties of the CsPbBr_2.5_I_0.5_ films. The excitation wavelength was 420 nm, and the PL spectrum peak was located at 720 nm with a full width at half maximum (FWHM) of about 20 nm. The increase in PL strength is due to surface passivation of the polymer [29]. There are a large number of defects in the perovskite film, resulting in exciton quenching and large leakage current. As shown in the SEM image, due to the addition of PAN, the pores on the surface of the film are significantly reduced to form a more uniform film. Notably, the PL intensity of the composite film is significantly higher than that of the pure perovskite film, which indicates that adding PAN can effectively improve the luminescence properties of perovskite film.

### 2.3. Performance of Red PeLEDs 

**Figure 5:**
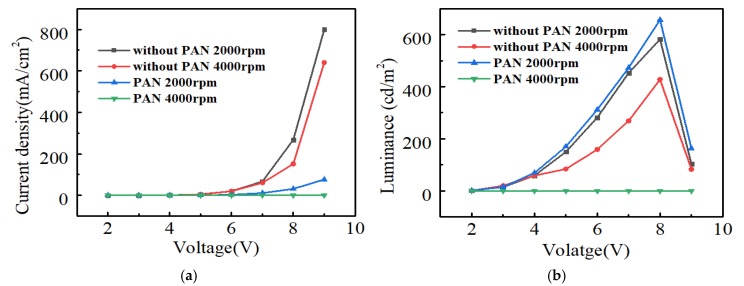
Current–luminance–voltage (I–L–V) characteristic curve of a red PeLED: (**a**) Current density–voltage (J–V) curve; (**b**) luminance–voltage (L–V) curve.

**Figure 6:**
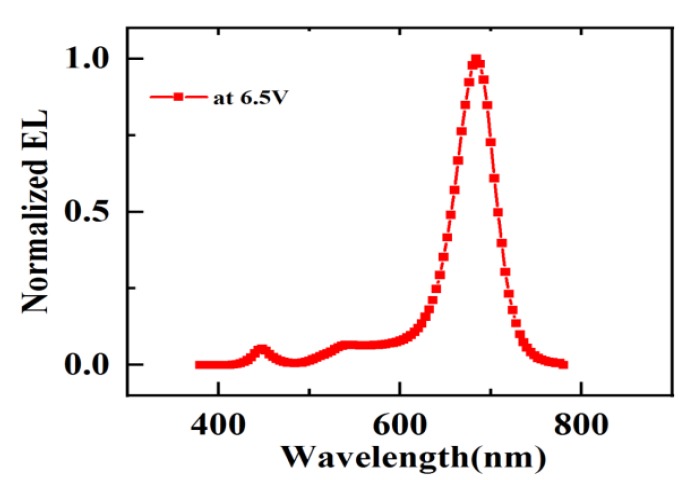
Electroluminescence spectra of a red PeLED.

In order to study the performance of PeLED devices composed of PAN:CsPbBr_2.5_I_0.5_ mixed films as light-emitting layers, we fabricated four groups of red PeLEDs at the following speeds: 2000 rpm/s without PAN, 2000 rpm/s with PAN, 4000 rpm/s without PAN, and 4000 rpm/s with PAN. The red PeLED current density–voltage (J–V) curve shown in Figure 5a exhibited typical diode characteristics of conventional PeLEDs; mainly, the current density increased exponentially with voltage. It is worth noting that the current density of the device decreased greatly due to the low conductivity of the PAN polymer [30]. Furthermore, the current density of the device at high speeds was slightly lower than that at lower speeds. Figure 5b shows the luminance–voltage (L–V) curves. The device with PAN fabricated at 2000 rpm/s exhibited a maximum luminance value of 657 cd/m^2^ at 8 V, whereas the device without PAN fabricated at 2000 rpm/s showed a maximum luminance value of 582 cd/m^2^ at 8 V. The brightness of the device increased by about 13%—such an effect proves that the PAN:CsPbBr_2.5_I_0.5_ composite film improves the performance of the device. In addition, the luminance of the device fabricated at 2000 rpm/s was higher than that fabricated at 4000 rpm/s. Adding PAN at 4000 rpm/s only showed very weak luminescence. This observation is explained as a consequence of the higher viscosity of the perovskite solution containing PAN, and the inferior morphology of the composite film at very high speeds. Since the thickness of the perovskite layer decreased at higher rotating speeds, a very thin perovskite film will also affect the performance of the device. We also spin-coated the PAN:CsPbBr_2.5_I_0.5_ solution at a low speed (1000 rpm/s). Since the viscosity of the solution was increased after the polymer was added to the perovskite precursor solution, the solution layer was thick, and there were more residual solvents. This resulted in a less uniform nucleation point, leaving a distinct pattern on the substrate after spin coating. The brightness of the device obtained on this basis is very uneven, and the performance is poor. Figure 6 shows the representative EL spectrum of a PeLED featuring a maximum intensity peak at 680 nm.

### 2.4. Performance of White PeLEDs

**Figure 7:**
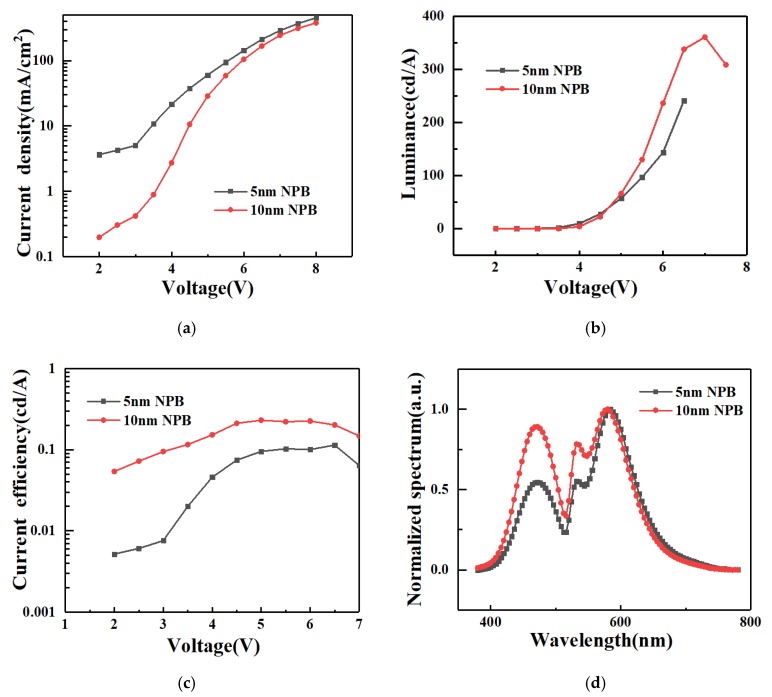
Current–luminance–voltage (I–L–V) characteristic curve and electroluminescence spectra of the white PeLED: (**a**) Current density–voltage (J–V) curve; (**b**) luminance–voltage (L–V) curve; (**c**) current efficiency–voltage (CE–V) curve; (**d**) electroluminescence spectra of the white PeLED device.

The current–voltage–luminance (J–V–L) curves for the white PeLED are shown in Figure 7. We tested the performance of the devices with different NPB thicknesses. The current density–voltage (J–V) curve is shown in Figure 7a. The growth of a 5 nm thick NPB PeLED exhibited a higher current density at low voltages compared to 10 nm thick NPB PeLED films. As the voltage increased, the difference in current density decreased. Luminance–voltage (L–V) curves and current efficiency–voltage (CE–V) curves are shown in Figure 7b,c, respectively. Applying a voltage exceeding the turn-on voltage (4V) of the device resulted in an exponential increase in luminance due to the concomitant increase in current density. The 10nm thick NPB PeLED reached a maximum luminance value of 360 cd/m^2^ at 7.5 V and a maximum current efficiency value of 0.2 cd/A. Above this voltage, the Joule heat generated at large currents caused deterioration of the device and a gradual decrease in luminance. Compared with the 10 nm thick NPB PeLED, the 5 nm thick NPB white PeLED had a maximum brightness value of 241 cd/m^2^ and a current efficiency of 0.1 cd/A, indicating that the thicker NPB made a certain improvement on device performance. The effect of NPB layer thickness on efficiency was due to the hole transport feature of NPB which has a blocking effect on electron transport, and the increase of NPB thickness contributed to carrier balance. However, as the NPB thickness increased, the device exhibited blue emission, as too many electron holes recombined in the NPB layer. Figure 7d shows the EL spectrum of white light-emitting PeLEDs. Under the condition of electroluminescence, the white light emitted by the device was a mixed blue, green, and red emission. The luminescence peak at 472 nm arose from the intrinsic blue light emission of the organic NPB layer, whereas the luminescence peaks at 532 nm (green light) and 608 nm (red light) originated from the intrinsic emission of the inorganic CsPbBr_3_ and CsPbBr_2.5_I_0.5_ materials, respectively. In addition, the devices with a 10 nm thick NPB layer exhibited stronger blue emission than those with a 5 nm thick layer. The CIE coordinate of the white PELED growing 10 nm NPB was (0.31, 0.36), which is consistent with pure white emission.

**Figure 8:**
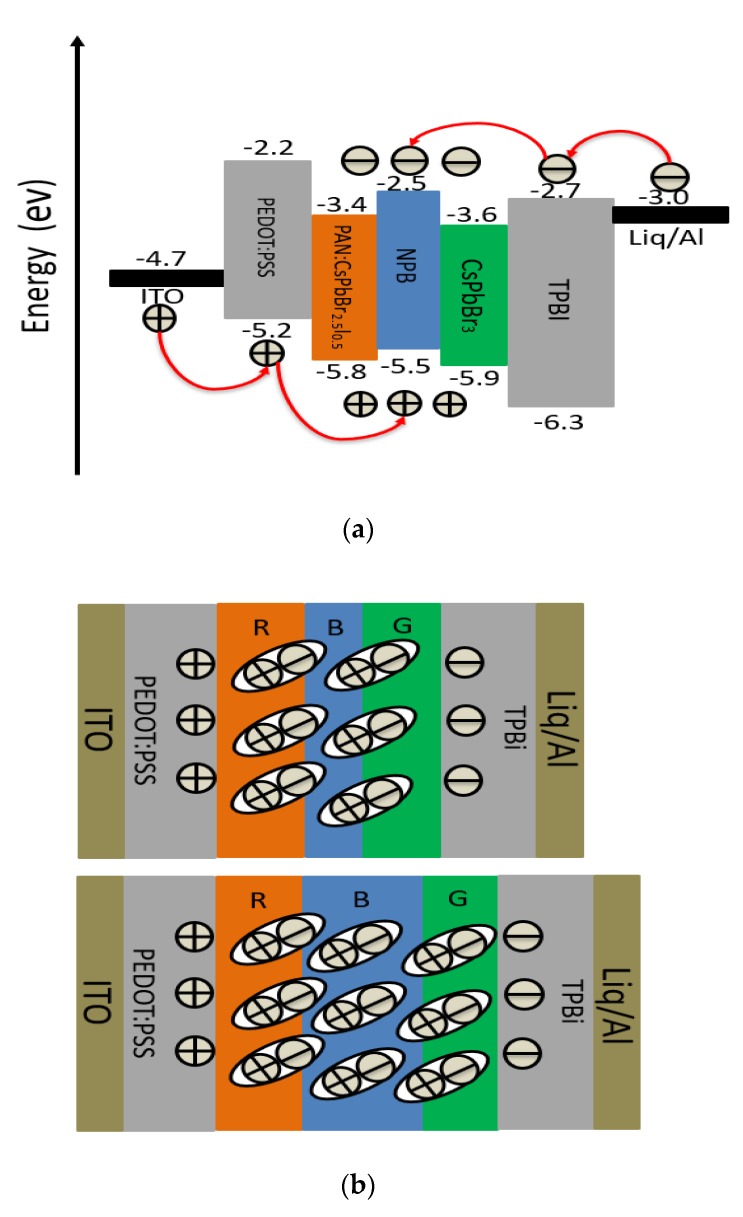
(**a**) Device energy level diagram of the white PeLED; (**b**) schematic diagram of the exciton distribution in the luminescent layer.

Figure 8a shows the working mechanism of the white PeLED based on the energy band diagram. Driven by the forward voltage, holes from the highest occupied molecular orbital (HOMO) of PEDOT:PSS and electrons from the lowest unoccupied molecular orbital (LUMO) of TPBi hopped to the interface of the luminescent layer to form excitons upon entering the luminescent layer. Overall, the 10nm thick NPB white light-emitting device exhibited significantly higher luminance and stronger blue emission than that of a device 5 nm in thickness (Figure 7). We speculate this is because as the thickness of NPB increases, the recombination region of electrons and holes also increases. In the case of 5 nm NPB, the number of excitons does not reach saturation, and as the thickness of NPB increases, there are more carrier recombinations in the luminescent layer which produces more excitons, resulting in higher luminance. A schematic diagram of the exciton distribution is shown in Figure 8b.

## 3. Materials and Methods

Cesium bromide (CsBr), lead bromide (PbBr_2_), iodine bromide (PbI_2_), 1,3,5-Tris(1-phenyl-1H-benzimidazol-2-yl)benzene (TPBi), 8-Hydroxyquinolinolato-lithium (Liq), and N,N’-Bis(naphthalen-1-yl)-N,N’-bis(phenyl)-benzidine (NPB) were purchased from Xi’an Polymer Light Technology Cory (Xi’an, China). Polyacrylonitrile (PAN, average M_w_ 150,000) were purchased from Sigma-Aldrich (Shanghai, China).

CsBr, PbBr_2,_ and PbI_2_ were dissolved in dimethyl sulfoxide (DMSO) to prepare 200 mg/mL of CsPbBr_2.5_I_0.5_ precursor solution. Polyacrylonitrile (PAN) was dissolved in DMSO to obtain a concentration of 10 mg/mL. The precursor solution was mixed with PAN solution at a volume ratio of 1.5:1 and stirred overnight.

Pre-patterned indium tin oxide (ITO) glass substrates were sequentially washed with cotton balls dipped in acetone, ethanol, and deionized water, and ultrasonically cleaned in acetone and ethanol for 15 min, respectively. The cleaned ITO glass substrate was transferred to a plasma cleaner for 30 min. PEDOT:PSS and isopropanol solutions were mixed in a 20:1 volume ratio, stirred, treated by ultrasound for 30 min, and then filtered. The solution was spin-coated onto an ITO substrate at 4000 rpm/s for 30 s. Then, the substrate was annealed in air at 150 °C for 20 min and transferred into a glove box. For the fabrication of red PeLEDs, a perovskite precursor solution was spin-coated onto the HTL (2000 rpm/s for 30 s) by a one-step spin coating method. The substrate was annealed in a nitrogen atmosphere at 80 °C for 10 min and then transferred into a vacuum thermal evaporation equipment connected to a glove box. Deposition of TPBi (70 nm), Liq (0.8 nm), and Al (140 nm) were carried out under a high vacuum atmosphere (~5 × 10^−4^ Pa). For the fabrication of white PeLEDs, PEDOT:PSS and CsPbBr_2.5_I_0.5_ precursor solutions were spin-coated onto ITO substrates, which were then annealed and transferred into a vacuum thermal evaporation equipment. A 10 nm thick NPB layer was deposited onto the substrate followed by another layer of CsPbBr_3_ synthesized by co-evaporation of CsBr and PbBr_2_. After that, TPBi (70 nm), Liq (0.8 nm), and Al (140 nm) were deposited successively.

The thickness and the deposition rate of each material layer were calibrated using an oscillating quartz thickness monitor. Photoluminescence (PL) spectra were measured using an RF-5301PC fluorescence spectrophotometer (Changchun, China). Electroluminescence (EL) spectra and CIE coordinates were measured using a PR655 spectra scan spectrometer (Changchun, China). Current–voltage–luminance characteristics were measured using an Agilent B290222A Precision Source/Measure Unit (Changchun, China) in the air at room temperature.

## 4. Conclusions

In this paper, we used a precursor solution containing an inorganic CsPbBr_2.5_I_0.5_ perovskite and PAN polymer. A composite PAN:CsPbBr_2.5_I_0.5_ film, prepared by a one-step method at low temperature, was used as the luminescent layer in red PeLEDs. The SEM and AFM results revealed a high coverage, compact, and uniform PAN:CsPbBr_2.5_I_0.5_ composite film that effectively reduced current leakage. The introduction of the polymeric material PAN played an important role in controlling the morphology of the perovskite film, which means that the PAN:CsPbBr_2.5_I_0.5_ mixed film can be used to make more efficient light-emitting devices. The device showed a maximum luminance value of 657 cd/m^2^ at 8V. Moreover, the exciton distribution of the perovskite and organic small molecule materials, used as the luminescent layer in white electroluminescent devices, was analyzed. The device featured a turn-on voltage of 4 V and a maximum luminance value of 360 cd/m^2^ at 7V. The color coordinate of the PeLED was (0.31, 0.36), which corresponds to an ideal white emission. Our innovative approach will encourage further developments in white perovskite LEDs.
